# Human Umbilical Cord MSC-Derived Exosomes Suppress the Development of CCl_4_-Induced Liver Injury through Antioxidant Effect

**DOI:** 10.1155/2018/6079642

**Published:** 2018-03-04

**Authors:** Wenqian Jiang, Youwen Tan, Mengjie Cai, Ting Zhao, Fei Mao, Xu Zhang, Wenrong Xu, Zhixin Yan, Hui Qian, Yongmin Yan

**Affiliations:** ^1^Liver Disease and Cancer Institute, School of Medicine, Jiangsu University, Zhenjiang, Jiangsu, China; ^2^The Affiliated Third Hospital of Zhenjiang, Jiangsu University, Zhenjiang, Jiangsu, China; ^3^Key Laboratory of Laboratory Medicine of Jiangsu Province, School of Medicine, Jiangsu University, Zhenjiang, Jiangsu, China

## Abstract

Mesenchymal stem cells (MSCs) have been increasingly applied into clinical therapy. Exosomes are small (30–100 nm in diameter) membrane vesicles released by different cell types and possess the similar functions with their derived cells. Human umbilical cord MSC-derived exosomes (hucMSC-Ex) play important roles in liver repair. However, the effects and mechanisms of hucMSC-Ex on liver injury development remain elusive. Mouse models of acute and chronic liver injury and liver tumor were induced by carbon tetrachloride (CCl_4_) injection, followed by administration of hucMSC-Ex via the tail vein. Alleviation of liver injury by hucMSC-Ex was determined. We further explored the production of oxidative stress and apoptosis in the development of liver injury and compared the antioxidant effects of hucMSC-Ex with frequently used hepatic protectant, bifendate (DDB) in liver injury. hucMSC-Ex alleviated CCl_4_-induced acute liver injury and liver fibrosis and restrained the growth of liver tumors. Decreased oxidative stress and apoptosis were found in hucMSC-Ex-treated mouse models and liver cells. Compared to bifendate (DDB) treatment, hucMSC-Ex presented more distinct antioxidant and hepatoprotective effects. hucMSC-Ex may suppress CCl_4_-induced liver injury development via antioxidant potentials and could be a more effective antioxidant than DDB in CCl_4_-induced liver tumor development.

## 1. Introduction

The imbalance between oxidant and antioxidant results in oxidative stress. Most chronic liver diseases, such as alcoholic liver disease, nonalcoholic fatty liver disease, liver fibrosis, and viral hepatitis, possess the increased oxidant stress [1]. Even in the progression of hepatocarcinogenesis, oxidative stress has been recognized as a key factor and increases the possibility of hepatocarcinogenesis [2]. Although antioxidants have been regarded as a good therapeutic strategy in consideration of the importance of oxidative stress in the pathological process of liver diseases, the research findings remain inconclusive and controversial. So searching for effective methods to control the oxidative stress is still on the way.

Mesenchymal stem cells (MSCs), with multilineage differentiation potential and self-renew ability, have given rise to interests in the potentials of repairing tissues. Increasing researches have taken advantage of MSCs to cell-based clinical trials for numerous diseases including liver diseases [3]. It is reported that MSCs exist in all tissues and can be isolated from bone marrow, adipose tissue, umbilical cord, and so on [4–6]. Human umbilical cord has been a prospective source of MSCs, with properties of proliferation and differentiation, lack of tumorigenicity, karyotype stability, and high immunomodulatory activity [7]. Our previous studies have successfully isolated the MSCs from human umbilical cord and demonstrated that human umbilical cord MSCs (hucMSCs) could ameliorate mouse hepatic injury and acute renal failure [8–11]. Early researches considered that the therapeutic mechanism of MSCs for repairing tissues was engraftment in injured tissues and differentiation into specific cells to replace necrotic or apoptotic cells [12, 13]. Recently, studies have suggested that mechanism of MSC in tissue repair may prefer secreting soluble factors to alter the tissue microenvironment rather than differentiation solely [14].

Exosomes (30–100 nm) are small membrane-bound vesicles derived from multivesicular bodies, which can be secreted by a wide variety of cells and contain proteins, mRNAs, and noncoding RNAs as cargos being transferred to other cells [15, 16]. Exosomes derived from MSC have shown to be beneficial to neurite outgrowth, neovascularization, and renal injury [17–19]. Our previous studies demonstrated that the protective effect of hucMSC-derived exosomes (hucMSC-Ex) on tissue repair including acute renal injury (AKI), cutaneous wound, and liver fibrosis [20–22] and even illuminated that hucMSC-Ex delivered GPX1 could promote the recovery of oxidatively injured liver [23]. However, whether hucMSC-Ex have any effects on liver injury development is not clear.

Bifendate, a synthetic intermediate of schisandrin C, was found to protect against drug-induced liver injury in animals and is now used clinically for the treatment of hepatitis [24]. In this study, we investigated the effects of hucMSC-Ex on liver injury development and explored the underlying mechanism preliminarily. We demonstrated that hucMSC-Ex could suppress CCl_4_-induced acute and chronic liver injury and liver tumor growth in mice. Furthermore, we compared the antioxidative and antiapoptotic effects of hucMSC-Ex with that of bifendate (DDB) in CCl_4_-induced acute liver injury.

## 2. Materials and Methods

### 2.1. Cell Culture

Fresh umbilical cords were harvested from informed, consenting mothers and processed within 6 h according to the experiment protocols approved by Jiangsu University (2012258) as previously described [8]. hucMSCs were cultured in L-DMEM containing 10% fetal bovine serum (FBS) (Bovogen, Australia) at 37°C with 5% CO_2_. Human normal hepatic L02 cells (Chinese Academy of Science) were maintained in RPMI 1640 containing 10% FBS (Bovogen, Australia) at 37°C with 5% CO_2._

### 2.2. Isolation and Characterization of Exosomes

Exosomes were isolated and purified as described previously [25]. Cell-conditioned medium with 10% FBS in which bovine exosomes and protein aggregates were removed by ultracentrifugation at 10000 ×g for 16 h at 4°C. Following 48 h culture, cell supernatants were collected and centrifuged at 2000 ×g for 20 min to remove cell debris and then centrifuged at 1000 ×g for 30 min using a 100 kDa molecular weight cutoff ultrafiltration membrane (MWCO) (Millipore, Billerica, Massachusetts, USA) to concentrate. After that, the concentrated supernatants were loaded upon a 30% sucrose/D_2_O cushion and ultracentrifuged at 100000 ×g for 2 h at 4°C. Exosomes were gathered from the bottom of the tube and washed with PBS for three times by centrifugation at 1000 ×g for 30 min using a 100 kDa MWCO (Millipore, Billerica, Massachusetts, USA). Exosomes were finally filtrated on a 0.22 *μ*m pore filter (Millipore, Billerica, Massachusetts, USA) and stored at −80°C. Concentration of concentrated exosomes was determined by nanoparticle tracking analysis (NTA) (NanoSight, Amesbury, U.K.). Exosomes were also identified by transmission electron microscopy (FEI Tecnai 12, Philips, Netherlands) for the morphology and the size and ImageStreamX Imaging Flow Cytometer (Amnis, WA, USA) for exosomal markers, CD9 and CD63.

#### 2.2.1. Animal Model and Exosome Injection

BALB/c female mice aged 4-5 weeks were purchased from the Laboratory Animal Center (Yangzhou University, China), and all experiment procedures were in accordance with the Chinese legislation regarding experiment animals. All the models were induced by intraperitoneal injection with 10% CCl_4_ dissolved in mineral oil, and the final dose was 0.3 ml CCl_4_/kg body weight. The normal group without any treatment was used as control (*n* = 6). Mice with liver tumor were induced with CCl_4_ every 3 days for 8 months and then treated with PBS (*n* = 6) or hucMSC-Ex (*n* = 6) via the tail vein. At 1 month after treatment, livers were harvested from sacrificed mice for further analysis. Mice for establishing liver fibrosis were treated with CCl_4_ every 3 days for 5 months and randomized into 2 groups for treating with PBS (*n* = 10) or hucMSC-Ex (*n* = 10). The dose of hucMSC-Ex was 6.4 × 109 particles per mouse diluted in 330 *μ*l PBS. One month later, mice were sacrificed to collect livers. Mice for acute liver injury were injected with CCl_4_ twice for an interval of 3 days. For the analysis of hucMSC-Ex in acute liver injury, mice were randomized into 2 groups for treating with PBS (*n* = 10) or hucMSC-Ex (6 × 10^10^ particles/kg; *n* = 10) by tail vein administration. For the comparison of antioxidative ability between hucMSC-Ex and DDB, mice were randomized into 2 groups for treating with hucMSC-Ex or DDB by intragastric administration at 24 h post-CCl_4_ injection. In each group, mice were divided into 3 groups randomly with the doses of hucMSC-Ex at 6 × 10^10^ particles/kg (*n* = 10), 1.2 × 10^11^ particles/kg (*n* = 10), and 2.4 × 10^11^ particles/kg (*n* = 10) or DDB at 8 mg/kg (*n* = 10), 16 mg/kg (*n* = 10), and 32 mg/kg (*n* = 10). At another 24 h, mice were sacrificed for further analysis.

### 2.3. CCl_4_-Induced L02 Cell Injury In Vitro

L02 cells were seeded in six-well plates at 1 × 10^5^ cells/well and were cultured with medium containing 0.1 mM CCl_4_/hucMSC-Ex (0 particles/ml, 4 × 10^8^ particles/ml, and 16 × 10^8^ particles/ml) for 24 hr. After hucMSC-Ex treatment, L02 cells were collected for further dectection.

### 2.4. Exosome Labeling and Tracing in Mice

According to the manufacturer's instructions, exosomes were incubated with the cross-linkable membrane dye, CM-DiR (Ruitai bio, Beijing, China), for 30 min at 37°C in the dark. After washing with PBS, the labeled exosomes were concentrated with a 100 kDa MWCO (Millipore, Billerica, Massachusetts, USA) at 1000 × g for 30 min at 4°C to remove nonbinding dye. Then CM-DiR-labeled exosomes were injected into CCl_4_-induced mice via the tail vein. After injection for 24 h, mice were imaged using a Maestro in Vivo Imaging System (CRI) to observe the distribution of exosomes.

### 2.5. Histopathological Staining

Liver tissues were fixed in 4% formaldehyde solution at room temperature overnight, embedded in paraffin, and cut into 4 *μ*m sections. The sections were stained with hematoxylin and eosin, Sirius red (Yeasen Biotechnology, Shanghai, China), and Masson trichrome (MT) (Gefan Biotechnology, Shanghai, China) in accordance with standard protocols. To analyze the extent of liver fibrosis, randomly picked fields of MT sections were captured from each animal.

### 2.6. Immunohistochemistry

Following deparaffinization and rehydration, the liver slides were steamed in citrate buffer (10 mM, pH 6.0) for 30 min for antigen retrieval and exposed to 3% hydrogen peroxide for 30 min for inhibiting endogenous peroxidase activity. Slides were then blocked in 5% bovine serum albumin for 1 h and incubated with the primary antibodies against SOX9 (Santa Cruz, Dallas, Texas, USA), 8-OHdG (Japan Institute for control of aging), activated caspase 3, Bax, and PCNA (both were from Bioworld, Louis Park, Minnesota, USA) overnight and then incubated with secondary antibody for 30 min at 37°C. Finally, slides were visualized with 3,3′-diaminobenzidine and counterstained with hematoxylin for microscopy examination (200x).

### 2.7. Western Blotting

hucMSC-Ex were lysed in RIPA buffer. Equal amount of protein was loaded and separated on a 12% SDS-PAGE gel. After electrophoresis, protein was transferred to PVDF membranes. The transferred membranes were blocked in 5% (*w*/*v*) skim milk for 1 h and incubated with the primary antibodies against CD9, CD63, Bcl2 (both were from Bioworld, Louis Park, Minnesota, USA), cleaved Casp3 (Santa Cruz, TX, USA), and GAPDH (KangCheng, Shanghai, China) at 4°C overnight and then incubated with HRP-conjugated goat anti-rabbit antibody for 1 h at 37°C. The signals were detected with a luminata™ crescendo Western HRP substrate (Millipore, Billerica, Massachusetts, USA) quantitated by a Molecular Dynamic Densitometer (Sage Creation Science) with LANE 1D software.

### 2.8. Lipid Peroxidation MDA Assay

Liver tissues were thawed on ice and then grinded into homogenate. Prepared homogenate was centrifuged at 400 ×g for 15 min to remove debris. The supernatant was then collected to measure MDA according to the manufacturer's instructions (Beyotime, Shanghai, China) and total protein concentration with a BCA assay kit (CWBIO, Beijing, China). MDA levels were normalized to milligrams of protein.

### 2.9. ELISA Assay

TGF-*β* levels in fibrotic liver tissues treated with PBS or hucMSC-Ex were determined using an ELISA kit (Boster, Wuhan, China) according to the manufacturer's instructions.

### 2.10. TUNEL Assay

The apoptotic cells in liver slides were measured by using an in situ cell apoptosis kit (Vazyme, Nanjing, China) according to the manufacturer's instructions.

### 2.11. Real-Time RT-PCR

Total RNA of mouse livers was extracted with the Trizol reagent according to the manufacturer's instructions (Invitrogen, Shanghai, China). The cDNA was synthesized using Super Scrispt™ RT kit according to the manufacturer's instructions (Invitrogen, Shanghai, China). The sequences of primers are shown in Table 1.

### 2.12. ROS Measurement

Cellular reactive oxygen species (ROS) of L02 cells was measured with a 2′-7′-dichlorofluorescein diacetate (DCF-DA) (Beyotime, Nantong, China) staining according to the instruction. Percentage and fluorescence intensity of DCF-positive cells were detected with the ImageStreamX Imaging Flow Cytometer (Amnis Corporation, Seattle, WA) and Olympus Fluorescent Microscope, respectively [23].

### 2.13. Statistical Analysis

Data are expressed as the means ± standard deviation (SD). Statistical significance was assessed by Student's *t*-test (two-tailed) using Prism software (GraphPad, San Diego, USA). *P* value < 0.05 was considered significant.

## 3. Results

### 3.1. hucMSC-Ex Suppressed the Development of Liver Tumor

**Figure 1:**
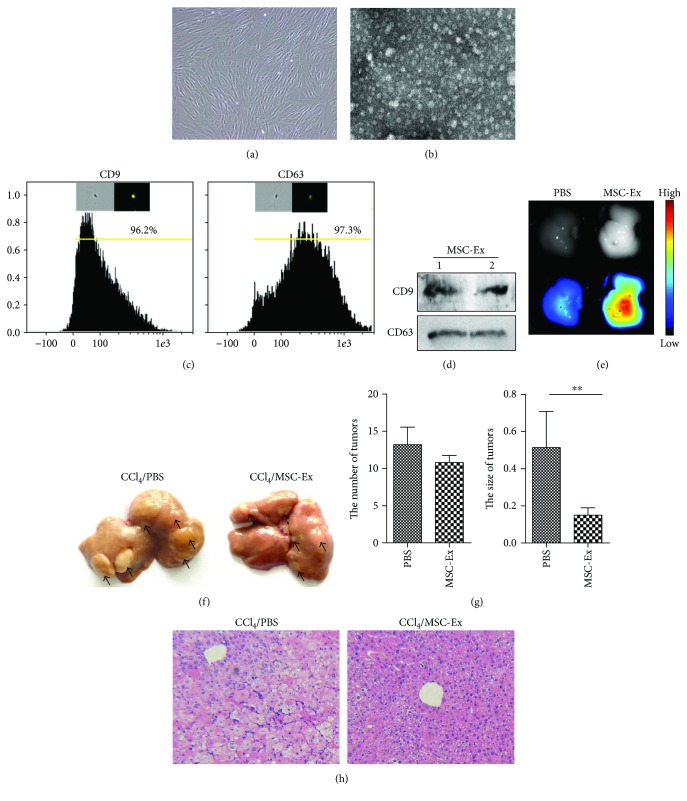
hucMSC-Ex suppressed CCl_4_-induced mouse liver tumor growth. (a) Morphological appearance of cultured hucMSCs. Original magnification 200x. (b) Identification of hucMSC-Ex with transmission electron micrograph. Scale bar 100 nm. (c) Imaging flow cytometer analysis of phenotypic markers of hucMSC-Ex. hucMSC-Ex were positive for CD9 and CD63. (d) Western blot assay indicated the positive expression of CD9 and CD63 proteins in hucMSC-Ex. (e) Distribution of CM-DiR labeled hucMSC-Ex in CCl_4_-induced hepatic carcinoma mice by in vivo fluorescent imaging. (f) Macroscopical observation of tumors in the liver surface of mice treated with PBS or hucMSC-Ex. The arrows indicated the tumors. (g) Analysis of the number and average size of tumors in the livers. Tumor size was significantly reduced in the hucMSC-Ex group (*n* = 6; ^∗∗^*P* < 0.01). (h) Representative images of H&E staining in the livers treated with PBS or hucMSC-Ex. Original magnification 100x.

To characterize hucMSC-Ex, primary hucMSCs were cultured in exosome-free media (Figure 1(a)). The exosomes were isolated and subjected to biochemical and biophysical analyses. Analysis of exosomes by transmission electron microscopy revealed that hucMSC-Ex were spheroid morphology with the diameter of 30–100 nm (Figure 1(b)). Imaging flow cytometry and Western blot analysis of exosomes demonstrated expression of the exosomal proteins CD9 and CD63 (Figures 1(c) and 1(d)).

To determine if hucMSC-Ex had any restrain effect on the development of liver tumor, CCl_4_-induced mouse liver tumor models were established and hucMSC-Ex was infused into the mouse intravenously. In vivo fluorescent imaging showed that CM-DiR labeled hucMSC-Ex mainly located at the liver at 24 h postinjection (Figure 1(e)). After treatment with CCl_4_ for 8 months, tumors were found on the surface of the livers. There is no difference in the number of liver tumors between the PBS group and the hucMSC-Ex group (Figure 1(f)). However, the size of tumors was obviously decreased in the hucMSC-Ex group compared with that in the PBS group (Figure 1(g); ^∗∗^*P* < 0.01). Hematoxylin and eosin (H&E) staining confirmed reduced areas of inflammation infiltration after hucMSC-Ex treatment (Figure 1(h)). These findings suggested that hucMSC-Ex could suppress the growth of liver tumor.

### 3.2. hucMSC-Ex Inhibited Oxidative Stress in Liver Tumor

**Figure 2:**
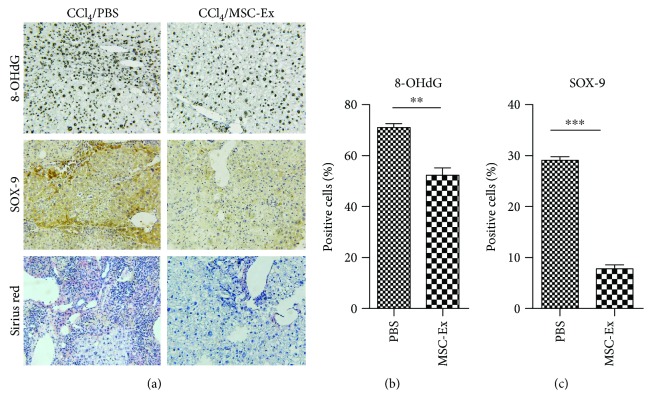
hucMSC-Ex reduced oxidative stress in mouse liver tumor. (a, b) The levels of 8-OHdG, SOX9, and collagen in mouse livers were measured by immunohistochemistry or Sirius red staining in liver tumor treated with PBS or hucMSC-Ex. Original magnification 200x. Compared with the PBS group, hucMSC-Ex significantly reduced production of oxidative stress marker 8-OHdG, SOX9 expression, and collagen deposition in liver tumor (*n* = 6; ^∗∗^*P* < 0.01, ^∗∗∗^*P* < 0.001).

Oxidative stress plays an essential role in the development of liver tumor. The hepatotoxicity of CCl_4_ is mainly about oxidative damage mediated by the production of reactive free radicals and results in damage to cells. To determine if there was any effect of hucMSC-Ex on liver tumor, the levels of oxidative stress product 8-OHdG were detected in the mice of the PBS or hucMSC-Ex group. Compared with the PBS group, 8-OHdG reduced significantly in the hucMSC-Ex group (Figures 2(a) and 2(b)). SOX9, an SRY-related HMG box transcription factor, is a progenitor/precursor cell marker of the liver expressed during embryogenesis, liver injury, and liver tumor [26]. Simultaneously, results of immunohistochemistry showed that SOX9 was downregulated in hucMSC-Ex treated mice (Figures 2(a) and 2(c)). Collagen deposition was also inhibited in the hucMSC-Ex group compared to the PBS group (Figure 2(a)). Thus, we preliminarily considered that hucMSC-Ex could reduce oxidative stress levels in liver tumor.

### 3.3. hucMSC-Ex Reduced Oxidative Stress and Inhibited Apoptosis in Liver Fibrosis

**Figure 3:**
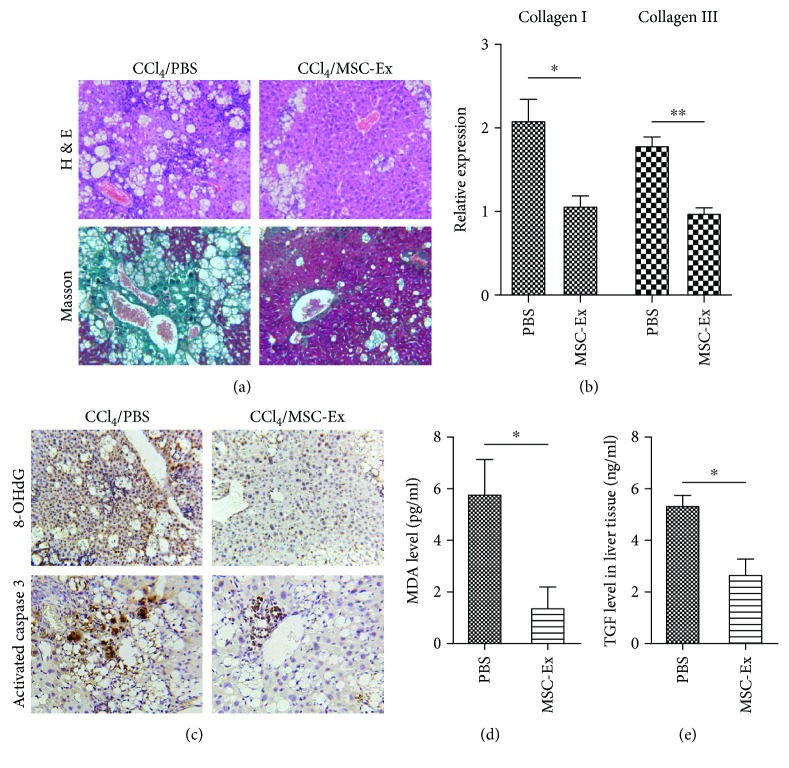
hucMSC-Ex reduced oxidative stress in mouse liver fibrosis. (a) Representative images of H&E and Masson staining of mouse fibrotic livers after PBS or hucMSC-Ex treatment. Reduced steatosis and collagen deposition were observed in the hucMSC-Ex group. Original magnification 200x. (b) Quantitative analyses of collagen I and III mRNA expression after hucMSC-Ex treatment (*n* = 10; ^∗^*P* < 0.05, ^∗∗^*P* < 0.01). (c) Immunohistochemistry analysis of 8-OHdG and activated caspase 3 after administration of PBS or hucMSC-Ex. Original magnification 200x. (d, e) MDA and TGF-*β* levels were measured in homogenates of a mouse fibrotic liver treated with PBS and hucMSC-Ex. The levels of MDA and TGF-*β* were inhibited by hucMSC-Ex compared with the PBS group (*n* = 10; ^∗^*P* < 0.05).

Liver tumors develop in the context of chronic liver diseases such as CCl_4_-induced liver fibrosis. We then investigated the effects of hucMSC-Ex- on CCl_4_-induced mouse liver fibrosis. To illustrate the antifibrotic effect, mice suffering liver fibrosis were handled with hucMSC-Ex, and PBS was used as controls. H&E staining showed that hucMSC-Ex treatment inhibited infiltration of inflammatory cells, hepatocyte apoptosis, and lobule destruction compared to PBS controls (Figure 3(a)). In the hucMSC-Ex group, obviously decreased areas of blue or green matrix which indicates collagen deposition were observed (Figure 3(a)). Moreover, the expression of collagen I and III detected by real-time RT-PCR decreased remarkably after hucMSC-Ex transplantation (Figure 3(b); ^∗^*P* < 0.05, ^∗∗^*P* < 0.01).

To confirm the abilities of hucMSC-Ex against oxidative stress and apoptosis in liver fibrosis, 8-OHdG and activated caspase 3 were detected by immunohistochemistry. Compared to PBS, hucMSC-Ex significantly inhibited activated caspase 3 and 8-OHdG production in mouse liver fibrosis models (Figure 3(c)). The levels of MDA and TGF-*β* in livers were also reduced in the hucMSC-Ex group (Figures 3(d) and 3(e); ^∗^*P* < 0.05). Thus, hucMSC-Ex may have potentials of oxidation resistance and antiapoptosis in liver fibrosis.

### 3.4. hucMSC-Ex Reduced Oxidative Stress and Inhibited Apoptosis in Acute Liver Injury

**Figure 4:**
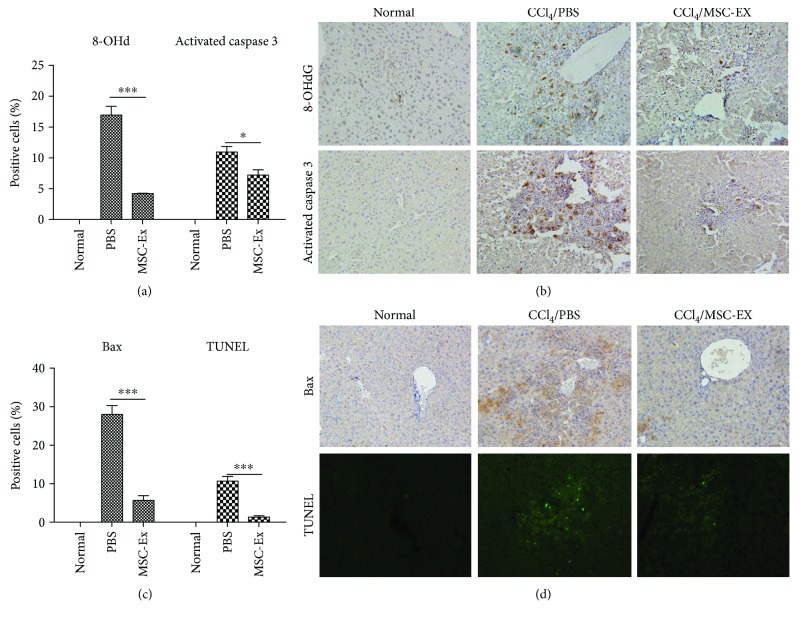
hucMSC-Ex reduced oxidative stress in acute liver injury. (a, b) Immunohistochemistry staining of 8-OHdG and activated caspase 3 in mouse livers. Reduced production of oxidative stress marker 8-OHdG and activated caspase 3 expression was detected in the hucMSC-Ex group (*n* = 10; ^∗^*P* < 0.05, ^∗∗∗^*P* < 0.001). Original magnification 200x. (c, d) Immunohistochemistry staining of Bax and TUNEL in mouse liver slides. Bax expression and cell apoptosis were decreased in the hucMSC-Ex group (*n* = 10; ^∗∗∗^*P* < 0.001). Original magnification 200x.

To further assess the antioxidative and antiapoptotic effects of hucMSC-Ex, they were injected intravenously into mouse models with acute liver injury induced by CCl_4_. The decrease of 8-OHdG production in an injured liver was found at 24 h after treatment with hucMSC-Ex (Figures 4(a) and 4(b); ^∗^*P* < 0.05, ^∗∗∗^*P* < 0.001). Activated caspase 3 and BAX have been shown to be involved in cell apoptosis. Then we explored the expression of activated caspase 3 and BAX. Results of Bax and activated caspase 3 staining showed cell apoptosis-associated gene expression was decreased after hucMSC-Ex treatment (Figures 4(a)–4(c); ^∗^*P* < 0.05, ^∗∗∗^*P* < 0.001). TUNEL staining also revealed that hucMSC-Ex treatment could inhibit the apoptosis in liver injury (Figures 4(c) and 4(d); ^∗∗∗^*P* < 0.001). Therefore, hucMSC-Ex can reverse oxidative stress-induced apoptosis in CCl_4_-induced acute liver injury.

### 3.5. Comparison of Antioxidative Ability between hucMSC-Ex and DDB in Acute Liver Injury

**Figure 5:**
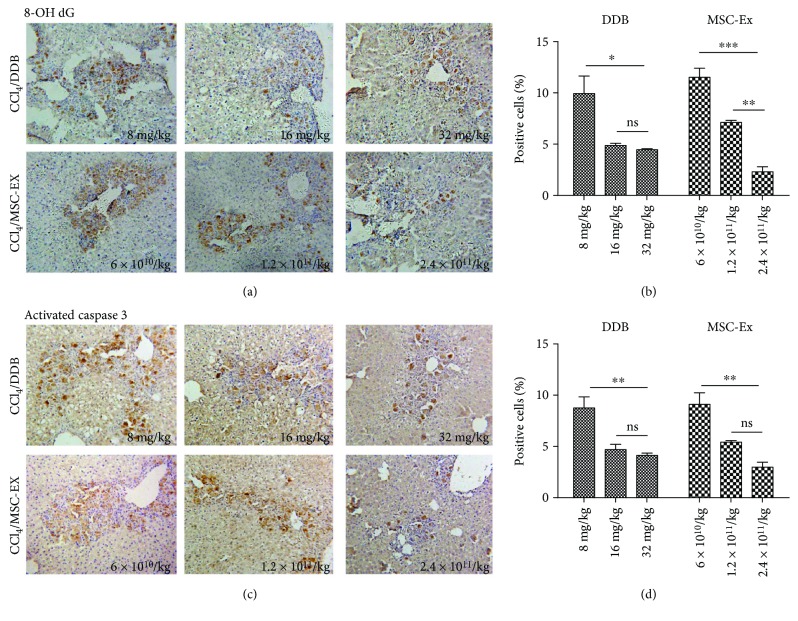
hucMSC-Ex exerted a more effective antioxidant than DDB in CCl_4_-induced liver injury. (a, b) Immunohistochemistry staining of 8-OHdG in mouse livers after treatment with DDB (8 mg/kg, 16 mg/kg, and 32 mg/kg) or hucMSC-Ex (6 × 10^10^ particles/kg, 1.2 × 10^11^ particles/kg, and 2.4 × 10^11^ particles/kg) (*n* = 10; ^∗^*P* < 0.05, ^∗∗^*P* < 0.01, and ^∗∗∗^*P* < 0.001). (c, d) Immunohistochemistry staining of activated caspase 3 in mouse livers after treatment with DDB (8 mg/kg, 16 mg/kg, and 32 mg/kg) or hucMSC-Ex (6 × 10^10^ particles/kg, 1.2 × 10^11^ particles/kg, and 2.4 × 10^11^ particles/kg) (*n* = 10; ^∗∗^*P* < 0.01). Compared with the PBS group, hucMSC-Ex (2.4 × 10^11^ particles/kg) exerted a more distinct inhibition effect in the expression of 8-OHdG and activated caspase 3. Original magnification 200x.

Bifendate (DDB) is frequently used in clinical therapy and can alleviate liver damage induced by CCl_4_. To identify the antioxidant ability of hucMSC-Ex, different doses of hucMSC-Ex (6 × 10^10^ particles/kg, 1.2 × 10^11^ particles/kg, and 2.4 × 10^11^ particles/kg) and DDB (8 mg/kg, 16 mg/kg, and 32 mg/kg) were given 24 h after administration of CCl_4_. We observed more integrated hepatic tissue structure and less hepatic lobule destruction in the hucMSC-Ex group at 1.2 × 10^11^ particles/kg compared with the DDB group at 16 mg/kg. Expression of 8-OHdG and activated caspase 3 was dose-dependently decreased in both the hucMSC-Ex group and the DDB group (Figures 5(a)–5(d); ^∗^*P* < 0.05, ^∗∗^*P* < 0.01, and ^∗∗∗^*P* < 0.001). Compared with the PBS group, hucMSC-Ex (2.4 × 10^11^ particles/kg) exerted a more distinct inhibition effect on the expression of 8-OHdG and activated caspase 3 (Figures 5(a)–5(d); ^∗^*P* < 0.05, ^∗∗^*P* < 0.01, and ^∗∗∗^*P* < 0.001).

**Figure 6:**
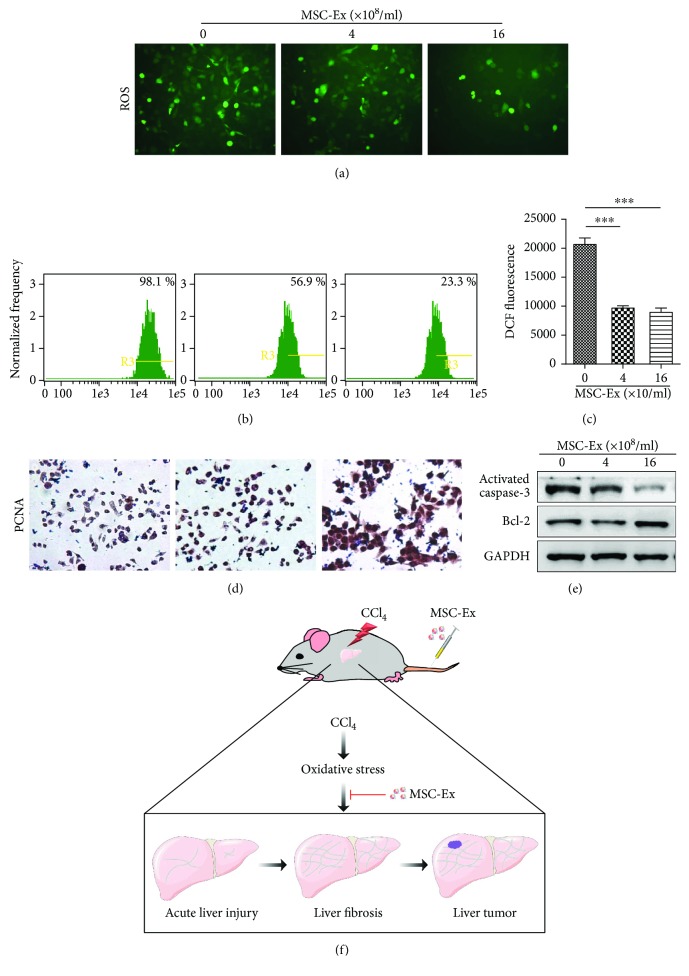
hucMSC-Ex inhibits oxidative stress in CCl_4_-injured L02 cells. (a) ROS production in CCl_4_-injured L02 cells treated with different doses of hucMSC-Ex (0 particles/ml, 4 × 10^8^ particles/ml, and 16 × 10^8^ particles/ml). Original magnification 200x. (b) Percentage of DCF-positive hepatocytes in hucMSC-Ex treated L02 cells using imaging flow cytometer. Data showed that hucMSC-Ex significantly decreased percentage of DCF-positive hepatocytes. (c) Fluorescence intensity of DCF-positive L02 cells was significantly decreased by hucMSC-Ex treatment. DCF fluorescent values are means ± SD. (*n* = 3; ^∗∗∗^*P* < 0.001). (d) Immunohistochemistry staining of PCNA in hucMSC-Ex-treated L02 cells. Percentage of PCNA-positive L02 cells and PCNA expression was enhanced in the hucMSC-Ex group compared with that in the PBS group. Original magnification 200x. (e) Western blot quantification of activated caspase 3 and Bcl2. hucMSC-Ex induced Bcl2 expression and inhibited activated caspase 3 expression in CCl_4_-injured L02 cells. (f) Experimental model design of hucMSC-Ex in CCl_4_-induced liver tumor development.

To further analyze the antioxidative effects of hucMSC-Ex *in vitro*, ROS level of the L02 cells were detected by using a DCF-DA probe. Results of imaging flow cytometry and fluorescence microscope showed that the percentage and fluorescence intensity of DCF-positive hepatocytes decreased after hucMSC-Ex treatment (Figures 6(a)–6(c); ^∗∗∗^*P* < 0.001). hucMSC-Ex treatment also promoted the expression of proliferating cell nuclear antigen (PCNA) (Figure 6(d)) and inhibited cell apoptosis-associated activated caspase 3 expression (Figure 6(e)). These findings demonstrated that hucMSC-Ex could be an effective antioxidant in CCl_4_-induced injury (Figure 6(f)).

## 4. Discussion

The capacities of MSCs including differentiation, immunomodulation, and bioactive molecule release determine the potentials to treat organ diseases induced by tissue injury or degeneration. A series of clinical trials was performed on patients with diseases such as liver cirrhosis and acute-on-chronic liver failure, which have demonstrated that hucMSC improved liver function and increased survival rates [27, 28]. Increasing evidences have demonstrated the paracrine effects of MSC on tissue repair; for example, Zhang et al. suggested that hucMSC rescue acute liver failure via paracrine actions to promote liver regeneration but not primarily attributing to differentiation into hepatocytes [29]. hucMSC-Ex consisting in hucMSC-conditioned medium contain proteins, mRNAs, noncoding RNAs, and other elements derived from the cells. Moreover, it has been reported that human MSC transplantation may promote tumor growth, while exosomes derived from hucMSC are supposed to be relatively safe [30, 31]. Given the similar functions of exosomes and their derived MSCs, it should be crucial for hucMSC-Ex on liver injury repair, simultaneously according to our previous studies [20, 32]. Even so, there is still no evidence stating any effect on liver injury development of hucMSC-Ex. Therefore, the present study was performed to illustrate the hepatoprotective role of hucMSC-Ex against liver injury development.

CCl_4_ is a hepatotoxic chemical and is frequently used to induce liver injury. After 8-month injection of CCl_4_, we successfully established a mouse model of liver tumor. Our data showed that after administration of hucMSC, the average volume of liver tumors was distinctly diminished. Although doses of 250 *μ*g exosomes were enough to ameliorate liver fibrosis and 200 *μ*g exosomes to improve wound healing, we still chose the high dose of hucMSC-Ex to transplant to observe the tumor-suppressive function with the consideration of severity of injured tissues in liver tumor.

Approximately 90% of liver tumors were developed in liver cirrhosis accompanied with chronic inflammation in which oxidative stress plays an influential role [33]. So far, oxidative stress has been the explanation of numerous liver disorders, and hence, we inferred that hucMSC-Ex exert tumor-suppressive function via inhibiting oxidative stress. Liver fibrosis, characterized by hepatic stellate cell activation and overdeposition of extracellular matrix, is the inevitable stage in the development of liver cirrhosis. Oxidative stress can also induce hepatic apoptosis [34]. We measured the high levels of 8-OHdG, activated caspase 3, and MDA in fibrotic livers, and hucMSC-Ex, as was expected, reduced the expression of these proteins. hucMSC-Ex also decreased TGF-*β* levels which are always induced in damaged livers and trigger hepatocyte destruction and hepatic stellate activation [35]. With regard to inflammation, hucMSC increased anti-inflammatory cytokines with the passage of time meanwhile it decreased proinflammatory cytokines, which indicated the relief of inflammatory reaction. Similar findings were observed in acute liver injury in vivo and in vitro that hucMSC-Ex exerted capacities of oxidation resistance and antiapoptosis. Wang had proved that exosomes derived from MSC gave play to hepatoprotective effects via activation of proliferation and regeneration [36]. In in vitro experiment, hucMSC-Ex presented obvious auxoaction of hepatocyte proliferation.

In spite of our understanding about the hepatoprotective effects of hucMSC-Ex, we still did not know well these potencies compared with other medicines. As is known to all, DDB, a synthetic intermediate of schisandrin C, is used to treat hepatitis with minimal side effects and is always regarded as positive control when exploring hepatoprotective actions [37, 38]. In the present study, we found that DDB presented the oxidation resistance and antiapoptotic potential with the increase of using dosage as well as hucMSC-Ex. However, there remained differences between the groups and the hepatoprotective effects of hucMSC-Ex were presented more distinctly than DDB.

In conclusion, we unraveled that hucMSC-Ex presented hepatoprotective activities through antioxidant defenses in the disease progression from initial liver injury to fibrosis and even to liver tumor. During the pathogenesis and regression of liver diseases, nevertheless, there are multifarious hepatotropic networks concerning tissue regulation and homeostasis [39], and hence, it is necessary to explore the further mechanism how hucMSC-Ex exert antioxidant activities in liver injury development.

## 5. Conclusions

hucMSC-Ex may suppress liver injury development via antioxidant potentials and could be a more effective antioxidant than DDB in liver injury.

## Figures and Tables

**Table 1:** Primers for real-time RT-PCR.

Genes	Primer sequence (5′-3′)	Annealing temperature (°C)	Product size (bp)
Mouse collagen I	For: TGAGACAGGCGAACAAGGTG	63	320
	Rev: GCTGAGGCAGGAAGCTGAAG		
Mouse collagen III	For: CTGGTCAGCCTGGAGATAAG	58	284
	Rev: ACCAGGACTACCACGTTCAC		
*β*-Actin	For: CACGAAACTACCTTCAACTCC	58	265
	Rev: CATACTCCTGCTTGCTGATC		
